# Biliary tract external drainage increases the expression levels of heme oxygenase-1 in rat livers

**DOI:** 10.1186/s40001-015-0152-2

**Published:** 2015-07-22

**Authors:** Lu Wang, Bing Zhao, Ying Chen, Li Ma, Er-Zhen Chen, En-Qiang Mao

**Affiliations:** Department of Emergency Intensive Care Unit, Shanghai Ruijin Hospital, School of Medicine, Shanghai Jiao Tong University, Shanghai, 200025 China

**Keywords:** Biliary tract external drainage, Heme oxygenase-1

## Abstract

**Background:**

Heme oxygenase-1 (HO-1) protects cells by anti-oxidation, maintaining normal microcirculation and anti-inflammatory under stress. This study investigated the effects of biliary tract external drainage (BTED) on the expression levels of HO-1 in rat livers.

**Methods:**

Biliary tract external drainage was performed by inserting a cannula into the bile duct. Sixty Sprague–Dawley rats were randomized to the following groups: sham 1 h group; BTED 1 h group; bile duct ligation (BDL) 1 h group; sham 6 h group and BTED 6 h group. The expression levels of HO-1 mRNA were analyzed using real-time RT-PCR. The expression levels of HO-1 were analyzed using immunohistochemistry.

**Results:**

The expression levels of HO-1 mRNA in the liver of the BTED group increased significantly compared with the sham group 1 and 6 h after surgery (*p* < 0.05).The expression levels of HO-1 in the BTED group increased significantly compared with the sham group 1 and 6 h after surgery. The expression levels of HO-1 mRNA in the liver in the BDL group decreased significantly compared with the sham group 1 h after surgery (*p* < 0.05).The expression levels of HO-1 in the BDL group decreased significantly compared with the sham group at this time.

**Conclusion:**

Biliary tract external drainages increase the expression levels of HO-1 in the liver.

## Background

Heme oxygenase-1 (HO-1) protects cells by anti-oxidation, maintaining normal microcirculation and anti-inflammatory under stress. Four decades of research have witnessed the HO-1 system continues to fascinate researchers across many areas of basic and clinical sciences [1, 2]. Bilirubin may play an negative feedback on the formation of HO-1 according to this theory. We speculate that biliary tract external drainage (BTED) may induce compensatory increase in HO-1 expression via blocking the enterohepatic circulation of bilirubin. Therefore, we explored effects of BTED on HO-1 expression in the liver.

## Methods

### Animal model

Sixty adult male Sprague–Dawley rats (250–300 g) were purchased from the Experimental Animal Center of Ruijin Hospital. Rats were housed and fed in accordance with the guidelines of the Committee on Care and Use of Laboratory Animal Resources approved by the Shanghai Jiao Tong University Medicine School Animal Care and Ethics Committee.

After a 1-week adaption period during which food and water were available ad libitum, rats were randomly divided into 5 groups: sham 1 h group; BTED 1 h group; bile duct ligation (BDL) 1 h group; sham 6 h group and BTED 6 h group. Rats were fasted overnight prior to experiments, but water was available ad libitum. Rats in the BTED group were intraperitoneally anesthetized with 3% sodium pentobarbital (0.2 ml/100 g). Laparotomies were performed after shaving and sterilization. Bile duct was exposed long enough for BTED. A catheter (inner diameter 0.4 mm; outer diameter 0.8 mm; length 20 cm) was inserted into the bile duct. The distal end of bile duct was ligated. The catheter was passed through the flank of rats to avoid bile passage into the gut and allow for the external collection of bile. In the BDL group, the bile duct was ligated. The abdomen was closed subsequently. Rats in the BDL group underwent pentobarbital anesthesia, laparotomy, bile duct ligation and suture. Rats in the sham group underwent pentobarbital anesthesia, laparotomy and suture. Twelve rats in every group were sacrificed at the point of setting time. Livers were collected.

### The expression levels of HO-1 messenger RNA (mRNA) in the liver

Liver scrapings from all animals were snap frozen and stored at −80°C for real-time RT-PCR. Total RNA was extracted from the liver using TRIzol reagent. Aliquots (2 μg) of total RNA were used to synthesize complementary DNA (cDNA). Purity and content of RNA was determined using ultraviolet spectrophotometry. A reverse transcription reaction was conducted in a mixture containing random primers, Revert Aid Reverse Transcriptase, RNase inhibitor, and dNTPs. The PCR reaction mixture was prepared using an SYBR Premix Ex Taq with specific upstream and downstream primers. The thermal cycling conditions were 10 s at 95°C for the initial denaturation step followed by 40 cycles of 95°C for 5 s and 60°C for 20 s in a real-time PCR system (7500, ABI, Foster, USA). The mRNA levels of HO-1 are expressed relative to the sham rats using the ΔΔCt method. The primers for HO-1 were 5′-ACCCCACCAAGTTCAAACAG-3′ and 5′-GAGCAGGAAGGCGGT-CTTAG-3′. The primers for β-actin were 5′-GCGCTCGTCGTCGACAACGG-3′ and 5′-GTGTGGTGCCAAATCTTCTCC-3′.

### Immunohistochemistry

Samples were fixed in 4% paraformaldehyde, embedded in paraffin and sectioned at 4 µm. Sections were mounted onto APES-coated slides, deparaffinized, rehydrated, incubated in 3% hydrogen peroxide to quench any endogenous peroxidase activity, and washed with distilled water and PBS for 5 min. Sections were placed in 3% citrate buffer to repair antigens. The buffer was heated to a temperature of 92–98°C using a microwave and maintained at that temperature for 10 min. The sections were cooled to room temperature. A 10% nonimmune goat serum was applied to eliminate nonspecific staining. Sections were incubated overnight at 4°C with optimally diluted primary antibody. Primary antibody used for immunohistochemistry was a rabbit polyclonal to HO-1 (1:200). Sections were washed with PBS and incubated with a broad-spectrum second antibody for 30 min, rewashed, and incubated with peroxidase-conjugated streptavidin for 15 min. Peroxidation activity was visualized via incubation with a peroxidase substrate solution (DAB kit). Sections were counterstained with hematoxylin.

### Reagents

TRIzol lysate was purchased from the Invitrogen Company (Frederick, USA), The revertAid first stand cDNA synthesis kit was purchased from the Thermo Company (Lithuania, EU), the Fluorescence quantitative RT-PCR kit was purchased from the Takara Company (Dalian, China), the HO-1 primers were synthesized by the Invitrogen Company (Shanghai, China), the anti-heme oxygenase 1 antibody was purchased from the Abcam Company (Cambridge, MA, USA), the immunohistochemistry kit was purchased from the Invitrogen Company (Frederick, USA).

### Statistics

Data were analyzed using SPSS 16.0 software. All data are expressed as mean ± SE of mean values, and results were compared using the unpaired Student’s *t* test and one-way analysis of variance followed by Tukey’s test. A *p* < 0.05 was considered to be statistically significant.

## Results

### Effects of BTED on the expression levels of HO-1 mRNA in the liver

**Figure 1:**
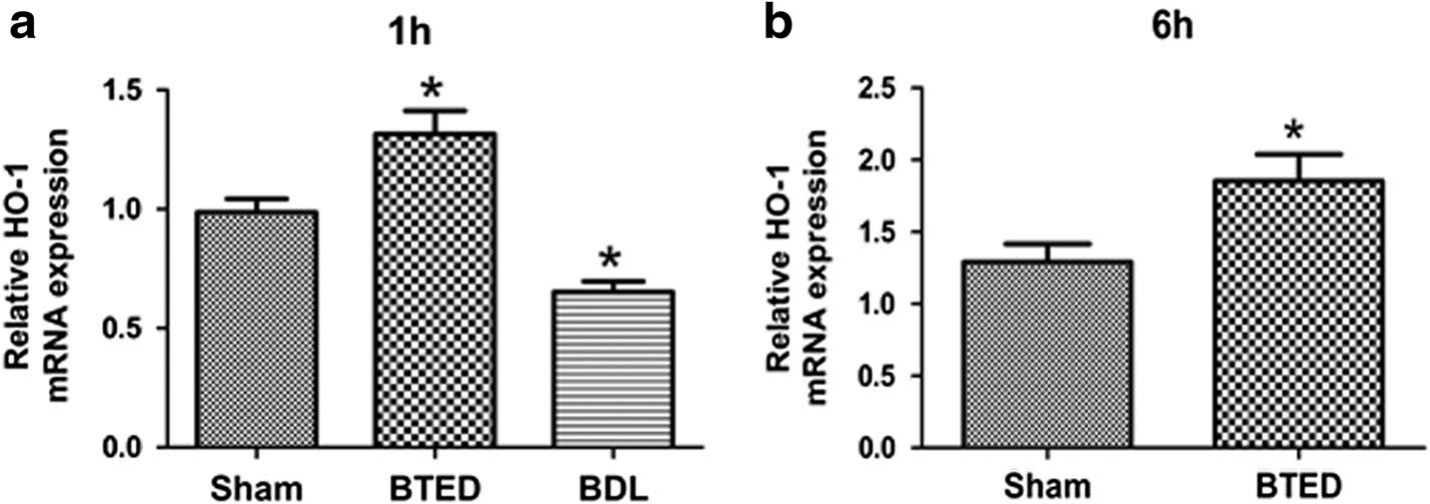
The expression levels of heme oxygenase-1 (HO-1) mRNA in the liver. **a** 1 h after operation; **b** 6 h after operation. Results are presented as mean ± SE of mean (n = 12). *p < 0.05, compared with the sham group. *BTED* biliary tract external drainage, *BDL* bile duct ligation.

The expression levels of HO-1 mRNA in the liver of the BTED group increased significantly compared with the sham group 1 h after surgery (*p* < 0.05) (Figure 1a). The expression levels of HO-1 mRNA in the liver of the BDL group decreased significantly compared with the sham group 1 h after surgery (*p* < 0.05) (Figure 1a). The expression levels of HO-1 mRNA in the liver of the BTED group increased significantly compared with the sham group 6 h after surgery (*p* < 0.05) (Figure 1b).

### Immunohistochemistry

**Figure 2:**
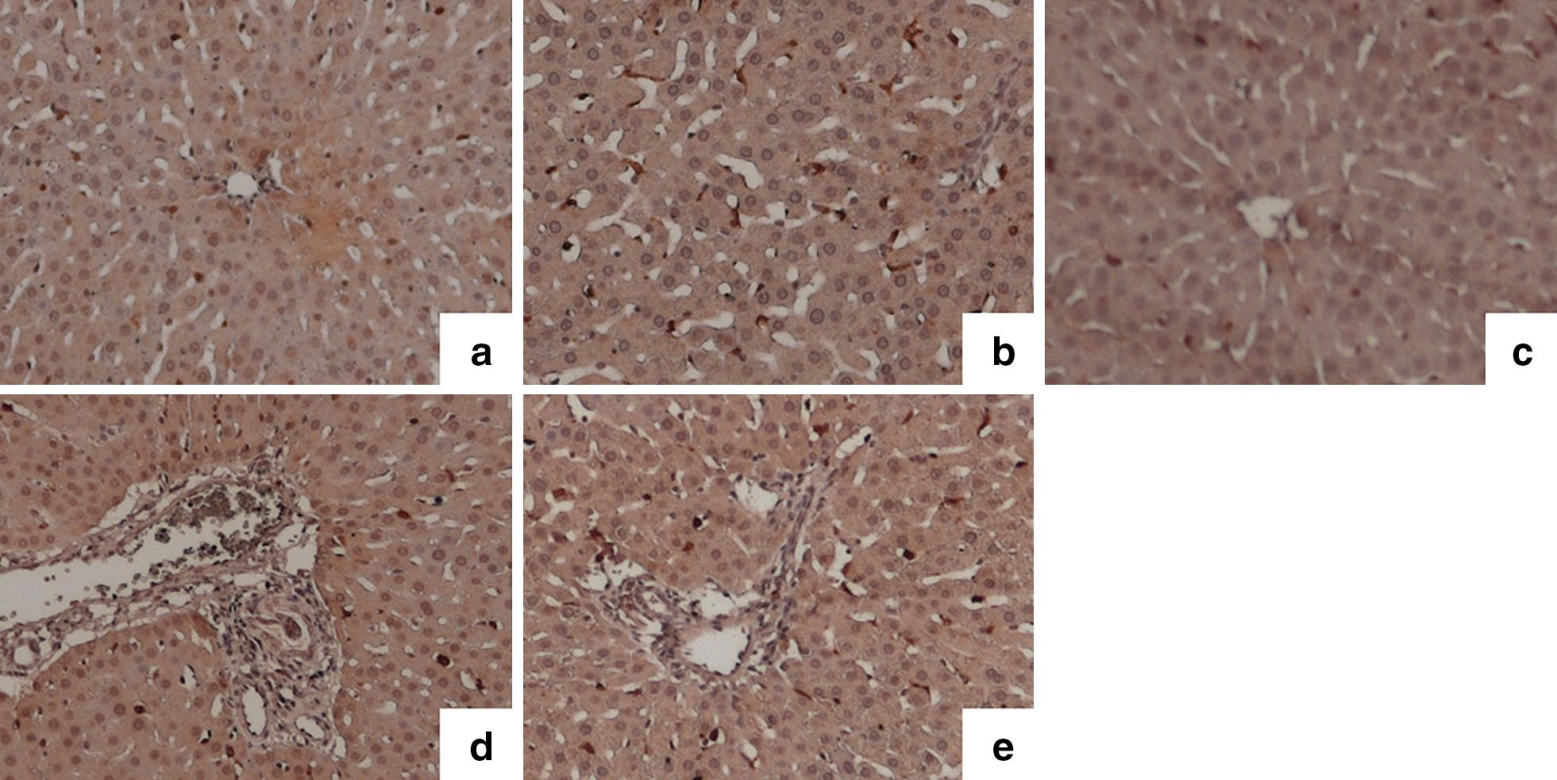
The expression levels of heme oxygenase-1 (HO-1) in the liver. **a** Sham 1 h group; **b** BTED 1 h group; **c** BDL 1 h group; **d** sham 6 h group; **e** BTED 6 h group. The expression levels of HO-1 in the liver increased significantly after BTED at 1 and 6 h after surgery. The expression levels of HO-1 in the liver decreased significantly after BDL at 1 h after surgery. *BTED* biliary tract external drainage, *BDL* bile duct ligation.

The expression levels of HO-1 in the liver of the BTED group increased significantly compared with the sham group 1 h after surgery (Figure 2a, b). The expression levels of HO-1 in the liver of the BDL group decreased significantly compared with the sham group 1 h after surgery (Figure 2a, c). The expression levels of HO-1 in the liver of the BTED group increased significantly compared with the sham group 6 h after surgery (Figures 2d, e).

**Figure 3:**
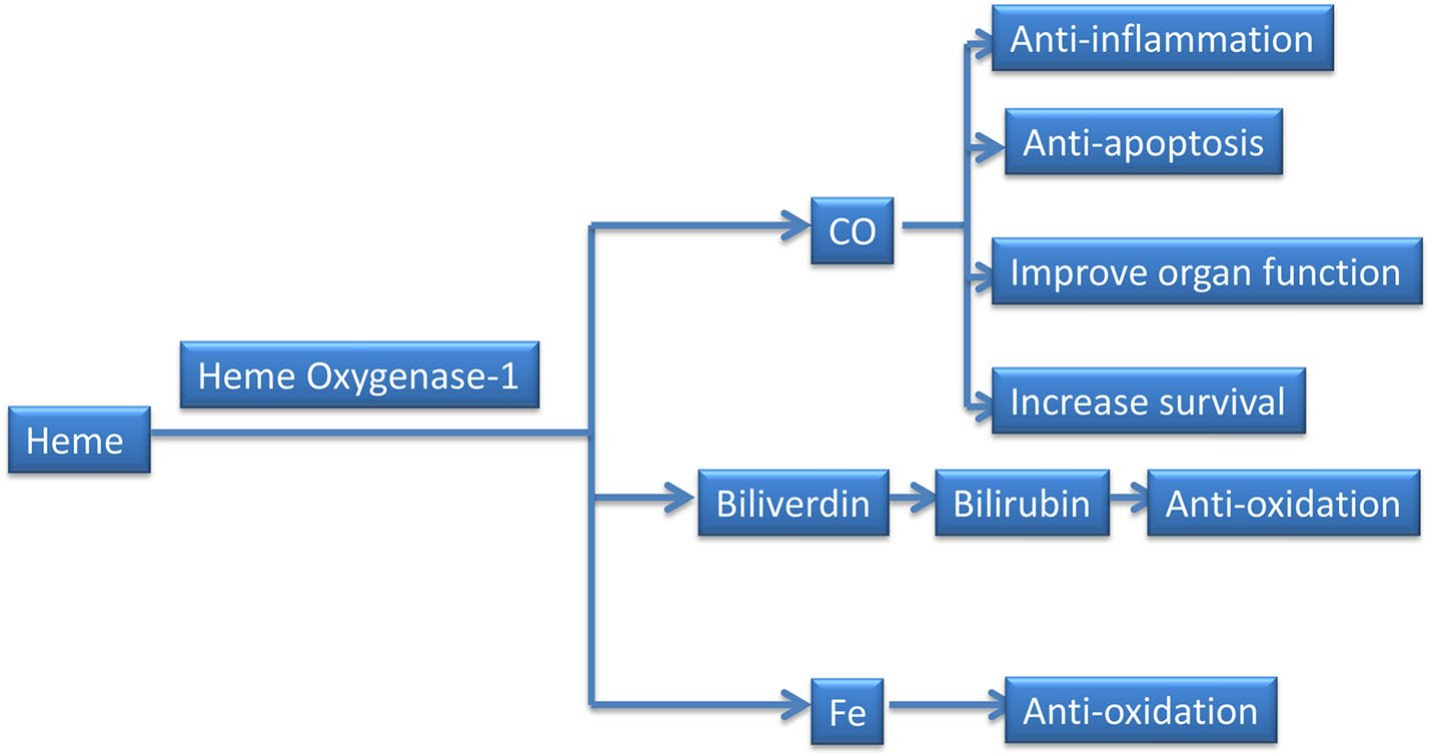
The three end-products of heme oxygenase-1 activity can protect cells against injury.

## Discussion

HO-1, a 32-kDa microsomal enzyme [3], catalyzes the rate-limiting step in oxidative degradation of heme to CO, biliverdin (soon reduced to bilirubin) and iron [4]. Since its discovery [5], studies have shown that HO-1 plays an important role in many modern medical disciplines, such as critical care [6–8], pulmonology [9–11], nephrology [12–14], gastroenterology [15–17], cardiology [18–20], neurology [21–23] and transplant immunology [24–26]. Sofalcone increases HO-1 in human umbilical vein endothelial cells and blocks endothelial dysfunction [27]. The polymorphism of the guanidinium thiocyanate repeats in the HO-1 promoter region is associated with the development of necrotizing acute pancreatitis [28]. Capsaicin induces HO-1 expression in kidney tissues and ameliorates cisplatin-induced renal dysfunction. Notably, the protective effects of capsaicin were completely abrogated by treatment with HO-1 inhibitor [29]. HO-1 expression protects the heart from acute injury [30].

The HO-1 system plays a vital role in anti-oxidative stress, anti-inflammation and regulation of cytokine expression. The analysis of the HO-1 gene knockout mice showed that HO-1 is an important molecule in systemic responses to stress. Endothelial cells are more susceptible to cytotoxicity induced by pro-oxidant stimuli in this case and produce more intracellular reactive oxygen species (ROS) when challenged with such stimuli [31–33]. HO-dependent protection is due to the reaction products of HO activity. CO, biliverdin and iron each contribute to the restoration of cellular homeostasis under inducing conditions [34, 35]. CO decreases proinflammatory cytokine production [36–39], reduces apoptosis [40–42], improves organ function [43, 44] and increases survival [45–47]. Biliverdin and bilirubin, the end bile pigments of heme degradation, protect cells against injury caused by oxidative stress in vitro [46, 48, 49]. Iron potentially acts as a catalyst of deleterious pro-oxidant reactions [50–52] (Figure 3).

Bilirubin may play a role via negative feedback on the formation of HO-1 according to theory. We speculate that BTED may induce compensatory increases in HO-1 expression in the liver by blocking the enterohepatic circulation of bilirubin. In this research, we found that the expression levels of HO-1 in the liver of the BTED group increased significantly compared with the sham group. The expression levels of HO-1 in the liver of the BDL group decreased significantly compared with the sham group. These results demonstrate that BTED may play important roles in anti-inflammation, anti-oxidative stress and regulation of cytokine expression by increasing the expression levels of HO-1. Specific roles of BTED in the treatment of diseases require further study.

## Conclusion

The expression levels of HO-1 mRNA and protein in the liver increased significantly from 1 h after BTED and this effect lasts for more than 6 h. The expression levels of HO-1 mRNA and protein in the liver decreased significantly 1 h after BDL.
